# CERV‐Score: A Hybrid Machine Learning Framework for Cervical Cancer Risk Prediction Using Integrated Clinical and Genomic Data

**DOI:** 10.1155/ijta/9913421

**Published:** 2026-05-06

**Authors:** Asma Mujahed Alanazi, Samia Dardouri

**Affiliations:** ^1^ Department of Computer Science, College of Computing and Information Technology, Shaqra University, Shaqra, Saudi Arabia, su.edu.sa; ^2^ InnoV′COM Laboratory—Sup′Com, University of Carthage, Tunis, Tunisia, ucar.rnu.tn

**Keywords:** cervical cancer, CERV-Score, clinical risk factors, decision support system, genomic integration, machine learning, predictive modeling, regression model, SMOTE

## Abstract

Cervical cancer remains a major global health burden, particularly in underserved populations where late diagnoses contribute to high mortality rates. Accurate, early risk prediction is essential for improving outcomes and guiding preventive care. In this study, we introduce CERV‐Score, a hybrid machine learning framework that advances prior approaches by combining structured clinical risk factors with recurrence‐based genomic markers to generate continuous, probabilistic risk scores rather than traditional binary classifications. This enables nuanced patient stratification into low, moderate, and high‐risk categories, providing clinicians with more actionable insights. Unlike previous models, CERV‐Score integrates genomic recurrence analysis identifying genes consistently expressed across multiple RNA‐seq samples to improve biological relevance and robustness. Additionally, we developed an interactive clinical‐genomic decision support tool that delivers real‐time, percentage‐based risk predictions and includes a gene lookup function, bridging clinical practice and molecular exploration in a single platform. The hybrid CERV‐Score model achieved high predictive performance (accuracy = 94.1*%*, F1 − score = 0.91, AUC = 0.94). Bootstrap resampling (1000 iterations) applied to the test predictions produced a 95% confidence interval for accuracy of 92.8%–95.4%, confirming the stability and robustness of the model′s performance. These results highlight the contribution of probabilistic scoring, recurrence‐driven genomic integration, and interactive visualization to enhance both accuracy and usability. By combining methodological innovation with practical clinical utility, CERV‐Score represents a meaningful step beyond existing hybrid models, laying the groundwork for more interpretable, personalized, and deployable cervical cancer risk prediction systems.

## 1. Introduction

Cervical cancer remains one of the most prevalent malignancies affecting women worldwide, highlighting the urgent need for improved early detection and prevention strategies. According to recent global statistics, approximately 660,000 new cases and 350,000 deaths were reported in 2022, making cervical cancer the fourth most common cancer among women [1]. The burden is particularly high in low‐ and middle‐income countries where access to prevention, screening, and treatment services remains limited. In Saudi Arabia, cervical cancer ranks as the eighth most common cancer among women, particularly affecting those aged 15–44 years [2]. Approximately 358 new cases and 179 deaths are reported annually, and more than 40% of cases are diagnosed at advanced stages [3, 4].

Conventional screening and diagnostic methods, including Pap smears, HPV testing, and colposcopy, remain essential tools for detecting cervical abnormalities. However, these approaches may suffer from false‐positive and false‐negative results and often require multiple clinical procedures and laboratory analyses, which can delay clinical decision‐making. Although screening and diagnosis are aimed at confirming the presence of disease through clinical testing, risk prediction focuses on estimating an individual′s probability of developing cervical cancer based on demographic, behavioral, and medical risk factors. Such predictive approaches can support earlier intervention by identifying individuals who may benefit from prioritized screening or closer monitoring.

Recent advances in artificial intelligence (AI) and machine learning (ML) have opened new opportunities for predictive modeling in healthcare. In particular, models that analyze clinical risk factors such as age, sexual behavior, smoking status, contraceptive use, reproductive history, and HPV infection have demonstrated potential for estimating disease risk. Nevertheless, models relying solely on clinical variables may lack sufficient biological depth. With the increasing availability of high‐throughput gene expression data, integrating genomic biomarkers with clinical information has emerged as a promising strategy to improve prediction accuracy and patient stratification.

In this study, we propose CERV‐Score, a hybrid ML framework designed to estimate the probabilistic risk of cervical cancer. The system combines structured clinical features with genomic knowledge derived from gene expression analyses. Using a random forest regression algorithm, the model generates an interpretable percentage‐based risk score through an interactive interface that supports both clinical risk assessment and gene‐level exploration. The overarching goal of this work is to develop an intelligent decision‐support tool that facilitates risk stratification, supports early intervention strategies, and contributes to improved outcomes for women at risk of cervical cancer.

## 2. Related Works

Recent research has increasingly focused on the application of AI and ML for predicting cervical cancer, with initial efforts primarily relying on structured clinical data or medical imaging. However, more recent developments have integrated genomic data to enhance prediction accuracy. Traditional models often use logistic regression or decision tree–based algorithms to analyze clinical variables, such as age, smoking status, and contraceptive use. For example, Belciug and Gorunescu [5] utilized support vector machines (SVMs) and decision trees for biopsy prediction in cervical cancer datasets, achieving moderate accuracy but with limited interpretability. Researchers have also explored techniques like feature selection and dimensionality reduction to improve model performance. Patil et al. [6], for instance, employed principal component analysis (PCA) alongside random forest classifiers to evaluate risk factors using publicly available cervical cancer data. Although their methods demonstrated effectiveness, they were confined to clinical features and lacked genomic integration.

In response to these limitations, hybrid models combining multiple data sources have garnered attention. Sharma et al. [7] proposed a model integrating clinical variables with selected gene expression profiles, resulting in significant improvements in diagnostic precision. Their findings highlighted the value of incorporating genomic data to enhance both sensitivity and specificity. High‐throughput RNA sequencing (RNA‐seq) has further facilitated the identification of differentially expressed genes (DEGs) associated with cervical cancer, showing promise as early diagnostic biomarkers. However, translating this complex molecular data into actionable clinical tools remains challenging. Additionally, many models remain limited to binary classification (e.g., cancer vs. noncancer) and lack probabilistic outputs, which are essential for risk stratification and informed clinical decision‐making. Recent advancements in AI and ML have significantly impacted the development of cervical cancer prediction models. Several studies have explored the use of clinical data, imaging, and genomic information to improve diagnostic accuracy and enable early intervention. For instance, Dweekat and Lam [8] employed an integrated system of PCA, genetic algorithms (GA), and multilayer perceptron (MLP) to enhance classification performance. Their method demonstrated promising results in handling complex, high‐dimensional data. Similarly, Yang et al. [9] conducted bioinformatics analysis to identify key genes associated with cervical cancer, focusing on genomic data analysis to identify biomarkers for early detection and prognosis. Agarwal et al. [10] developed a curated database of genes involved in cervical cancer (CCDB), providing valuable resources for researchers exploring the genetic aspects of cervical cancer. Ijaz et al. [11] introduced a data‐driven cervical cancer prediction model using outlier detection and oversampling methods to handle imbalanced data. This model effectively improved prediction accuracy, particularly in the context of disproportionate class distributions commonly found in medical datasets. Uddin et al. [12] employed an ensemble ML approach using hybrid feature selection methods, showing improvements over single‐algorithm approaches. Tang et al. [13] proposed a hybrid strategy incorporating conditional generative adversarial networks (CTGAN) to generate synthetic data, addressing data scarcity issues in cervical cancer diagnosis. Similarly, Lilhore et al. [14] utilized causal analysis and ML techniques to develop a hybrid model focused on identifying causal relationships between clinical and genomic factors. Ahishakiye and Kanobe [15] optimized cervical cancer classification using transfer learning with deep Gaussian processes and SVM, enhancing performance by leveraging pretrained models. Rahimi et al. [16] conducted a systematic review on ML algorithms for predicting cervical cancer survival, evaluating various models and their clinical potential. These studies provide crucial insights for research on hybrid ML models in cervical cancer prediction. Notably, Geeitha et al. [17] used bidirectional recurrent neural networks (RNNs) for cancer recurrence and survival prediction, demonstrating the value of integrating temporal clinical data. Xu et al. [18] proposed a prognostic risk model based on immune checkpoint HLA‐G–driven genes, emphasizing the importance of genomic data in risk prediction. Li et al. [19] demonstrated a multivariable model that combines clinical and SNP data for early detection, whereas Mehmood et al. [20] explored various ML techniques for cervical cancer detection. Recent works have also incorporated decision tree and Naïve Bayes classifiers, as seen in Patel et al. [21], which applied these techniques on the UCI Cervical Cancer Risk Factors dataset, achieving moderate predictive accuracy. Baloch et al. [22] achieved similar results with SVMs and logistic regression but faced challenges related to class imbalance. More recent advancements have explored ensemble learning methods, such as those by Yassin et al. [23], who integrated random forest and gradient boosting models to improve precision and recall in imbalanced datasets. However, these models often lack genomic context and real‐time interactivity, limiting their clinical application. Additionally, some studies, like those by Tang et al. [24], have used RNA‐seq profiles and deep learning to classify cervical cancer subtypes, underscoring the potential of transcriptomic features for personalized diagnostics. Yet, these models often require large‐scale datasets, limiting their feasibility in resource‐constrained settings. Collectively, these studies illustrate the growing importance of hybrid models that combine clinical, genomic, and temporal data to enhance the accuracy and effectiveness of cervical cancer prediction and diagnosis. Although these models represent significant progress, many focus solely on either clinical or genomic domains, or rely on binary classification. In contrast, CERV‐Score offers a hybrid regression‐based approach that not only integrates both clinical and genomic inputs but also provides interpretable, continuous risk scores, enhancing its clinical utility in real‐time decision support.

## 3. Materials and Methods

### 3.1. Dataset Description

To develop an effective hybrid predictive model, we integrated publicly available clinical and genomic datasets that provide complementary insights into cervical cancer risk. The clinical component was sourced from the UCI Machine Learning Repository (“Cervical Cancer Risk Factors”), comprising 858 patient records containing demographic, behavioral, and medical history variables such as age, number of sexual partners, age at first intercourse, smoking status, contraceptive use, and history of sexually transmitted infections. To enhance the robustness of the genomic component and avoid reliance on a single small dataset, we expanded beyond the initial GSE253690 collection by incorporating two additional Gene Expression Omnibus (GEO) datasets, GSE6791 and GSE63514, which include RNA‐seq and microarray expression profiles from cervical cancer samples and matched controls. This multidataset integration increases biological diversity, reduces dataset‐specific bias, and provides a more reliable foundation for the identification of DEGs used in the hybrid model. Table 1 describes the datasets used in this study, including their sources, data types, sample sizes, and platform technologies.

**Table 1:** Summary of datasets used in the CERV‐Score framework.

Dataset	Source	Data Type	Samples	Platform
Cervical cancer risk factors	UCI Machine Learning Repository	Clinical	858 patients	Structured clinical dataset
GSE253690	GEO	RNA‐seq	Cervical cancer samples	RNA sequencing
GSE6791	GEO	Gene expression	Cervical tissue samples	Microarray
GSE63514	GEO	Gene expression	Cervical lesion samples	Microarray

The clinical dataset was obtained from the UCI Machine Learning Repository and contains 858 patient records with demographic, behavioral, and medical risk factors associated with cervical cancer. In addition, three genomic datasets (GSE253690, GSE6791, and GSE63514) were collected from the GEO database. These datasets include gene expression profiles derived from RNA‐seq and microarray platforms. Integrating multiple datasets provides greater biological diversity, reduces dataset‐specific bias, and strengthens the robustness of genomic marker identification within the CERV‐Score framework.

### 3.2. Data Preprocessing

To ensure data quality and optimize model performance, several preprocessing steps were applied to both clinical and genomic datasets.

• Clinical data processing: Clinical data cleaning involved handling missing values by converting them to NaN and applying appropriate strategies such as median or mode imputation. Rows with excessive missing values were removed when necessary. Categorical and binary variables were numerically encoded, and continuous variables were standardized using the StandardScaler to ensure consistent feature scaling across the dataset.
• Genomic data processing: To improve robustness and avoid reliance on a single dataset, genomic data were collected from multiple GEO datasets (GSE253690, GSE6791, and GSE63514). DEGs were identified using established statistical pipelines. Specifically, DESeq2 was applied to RNA‐seq datasets, whereas limma was used for microarray datasets, following standard gene expression analysis practices. Genes were considered significant if they satisfied the criteria FDR < 0.05 and |log_2_ fold change| > 1. These thresholds ensured statistical reliability and biological relevance of the selected markers.

To further support exploratory analysis within the interactive tool, an additional filtering step was applied to identify genes consistently expressed across multiple cancer samples. Genes exhibiting expression in at least two of the three available RNA‐seq cancer samples were considered recurrently expressed and included in the gene‐lookup module of the decision‐support interface. Importantly, this recurrence‐based filtering was used only for exploratory gene validation within the interface and not as part of the statistical differential expression analysis pipeline.

### 3.3. Proposed Model

To estimate the probabilistic risk of cervical cancer, we developed a regression‐based ML model using structured clinical features. The entire clinical dataset was split into training (70%), validation (15%), and independent testing (15%) subsets to ensure reliable generalization. In addition, 10‐fold stratified cross‐validation was applied on the training set to enhance robustness and reduce bias associated with single‐split evaluations.

#### 3.3.1. Handling Class Imbalance

Because the ground‐truth outcome in the UCI dataset is binary (cervical cancer: yes/no), the data exhibited substantial class imbalance. To address this issue, several resampling strategies were evaluated, including SMOTE, ADASYN, and random undersampling. To prevent data leakage and ensure fair model evaluation, resampling techniques were applied only within the training folds during cross‐validation, whereas validation and test sets were kept untouched. SMOTE was selected for the final model because it provided the most balanced representation of the minority class during training.

#### 3.3.2. Model Selection and Optimization

A Random Forest Regressor was selected due to its resilience to overfitting, capacity to model nonlinear interactions, and strong empirical performance in clinical risk modeling. Hyperparameters were optimized using GridSearchCV, with the following parameter search space:

• n_estimators: [100, 300, 500, 1000]
• max_depth: [5, 10, 20, None]
• min_samples_split: [2, 5, 10]
• min_samples_leaf: [1, 2, 4]
• bootstrap: [true, false]

This exhaustive search ensured a balanced exploration of model complexity and computational efficiency.

#### 3.3.3. Hybrid Clinical–Genomic Framework

The proposed framework integrates two complementary components. First, the predictive model utilizes structured clinical variables obtained from the UCI Cervical Cancer Risk Factors dataset. Second, statistically validated genomic markers were identified through differential expression analysis across three GEO datasets (GSE253690, GSE6791, and GSE63514). For genomic analysis, DESeq2 was applied to RNA‐seq datasets, whereas limma was used for microarray datasets, and DEGs were selected using the thresholds FDR < 0.05 and |log_2_FC| > 1.

To maintain interpretability and avoid overfitting given the modest genomic sample sizes, the identified DEGs were not used directly as predictors in the random forest regression model. Instead, these genomic markers were incorporated into the gene‐lookup module of the interactive decision‐support interface, allowing users to explore biologically relevant genes associated with cervical cancer. This design preserves the transparency of the clinical prediction model while providing genomic context for biological interpretation within the tool.

#### 3.3.4. Prediction and Evaluation

Although the ground‐truth outcome is binary (cervical cancer: yes/no), a regression framework was intentionally adopted so that CERV‐Score generates a continuous and interpretable probability value between 0 and 1, rather than a discrete class label. This approach enables more refined clinical risk stratification, allowing patients to be categorized into varying risk levels rather than a simple binary outcome.

To comprehensively evaluate model performance, both regression and classification metrics were employed. For the regression evaluation, mean absolute error (MAE) and the coefficient of determination (R‐squared [*R* ^2^]) were used to measure how accurately the model estimates the underlying risk probability. To facilitate comparison with conventional classification‐based cervical cancer prediction models, predicted probabilities were subsequently thresholded to produce binary predictions, enabling the calculation of accuracy, precision, recall, F1‐score, and AUC‐ROC.

In addition, calibration curves and decision curve analysis (DCA) were used to assess the clinical reliability and potential decision‐making value of the predicted probabilities. Finally, the statistical significance of performance improvements over baseline models was evaluated using paired *t*‐tests and Wilcoxon signed‐rank tests.

#### 3.3.5. Interactive Prediction Tool

An interactive prediction tool was created using ipywidgets within a Jupyter Notebook environment, allowing real‐time input of selected clinical risk factors. This tool provides a predicted percentage risk score for cervical cancer based on the input data. Additionally, it includes a gene lookup feature that verifies whether a user‐input gene is part of the set of highly recurrent genes identified in cancer samples. This dual‐function system facilitates both clinical risk evaluation and gene‐level validation, improving usability and supporting personalized medicine practices by integrating clinical and genomic insights.

**Figure 1:**
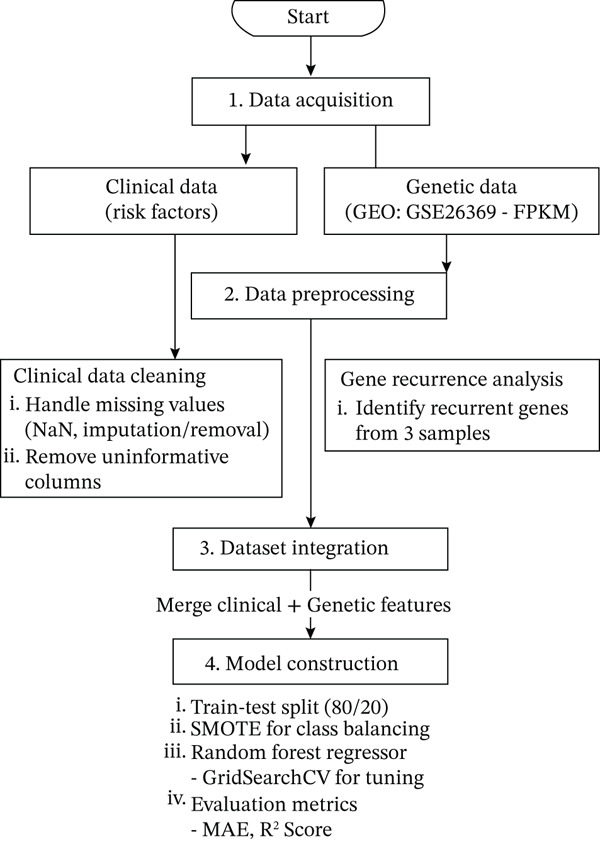
Overview of the proposed CERV‐Score framework.

The proposed model is illustrated in Figure 1, which visually represents the structure and workflow of the CERV‐Score tool. This figure highlights the integration of clinical and genetic data, the process of generating the continuous risk score, and the intuitive interface for risk categorization into low, moderate, and high levels. It provides a clear depiction of how the model supports clinical decision‐making and enhances interpretability, helping healthcare professionals better assess and manage cervical cancer risk.

**Figure 2:**
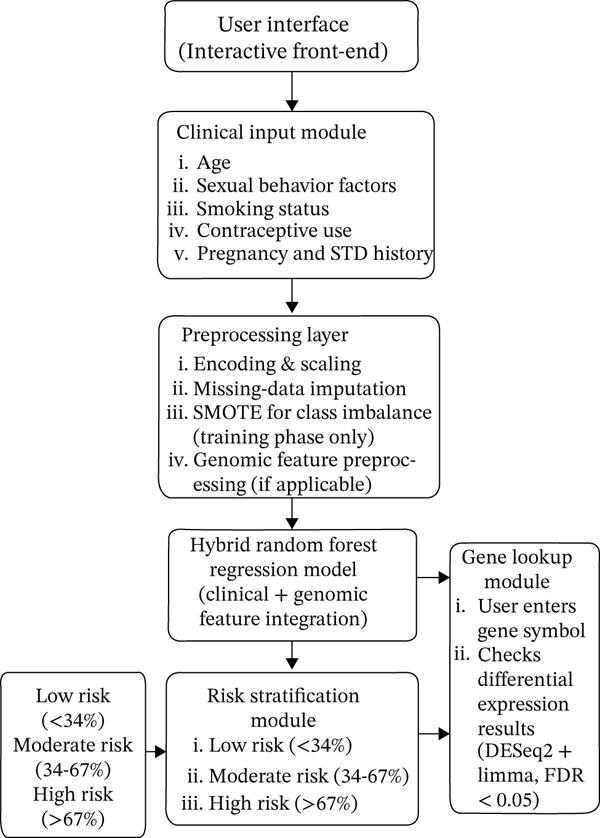
Architecture of the CERV‐Score interactive prediction tool.

To enhance transparency and illustrate the translational design of the system, we incorporated an architecture diagram that summarizes the workflow of the interactive tool. As shown in Figure 2, the interface accepts structured clinical inputs (e.g., age, sexual behavior variables, smoking status, and contraceptive use), which are passed to the trained random forest regression model to generate a continuous risk probability. The tool then categorizes the risk into low, moderate, or high levels based on predefined thresholds. In parallel, the gene lookup module allows users to query specific gene names and confirm whether they belong to the set of statistically validated DEGs. The interface is implemented using ipywidgets in Jupyter Notebook, providing real‐time prediction, intuitive layout, and dual clinical–genomic functionality tailored for clinician use.

**Figure 3:**
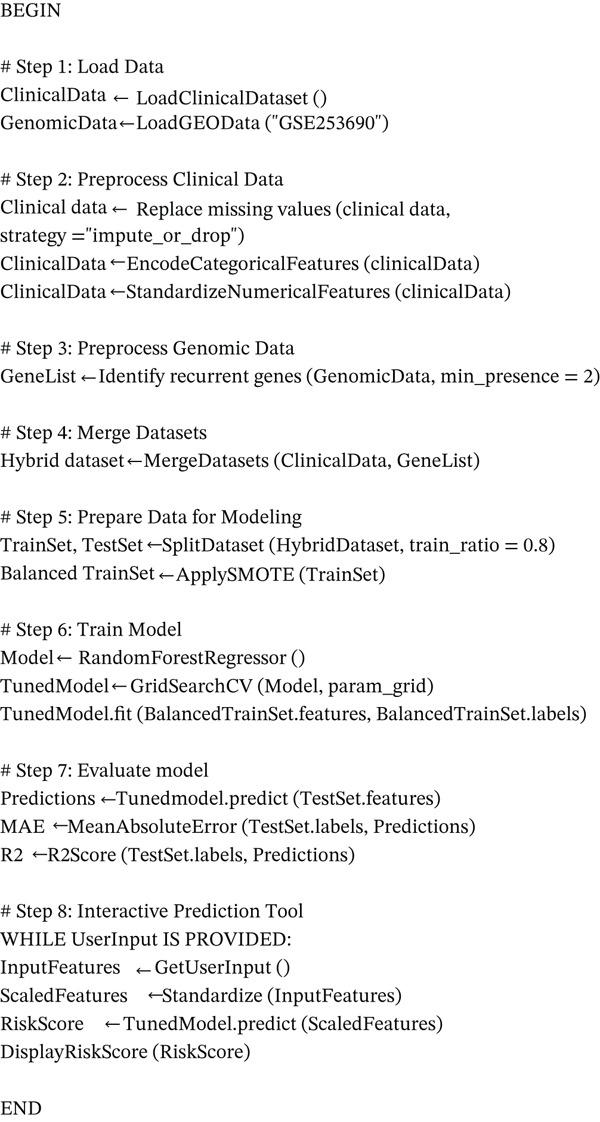
Pseudocode of proposed model.

The pseudocode is illustrated in Figure 3, where it outlines the key steps involved in the CERV‐Score model′s development and operation. This figure demonstrates the process flow, from data preprocessing and feature encoding to model training, risk score prediction, and final output generation. It also includes the handling of class imbalance using SMOTE and the application of Random Forest Regressor for accurate risk estimation. The pseudocode provides a high‐level abstraction of the model’s workflow, facilitating a clearer understanding of the underlying methodology and the interactions among its components.

#### 3.3.6. Real‐World Deployment and Telemedicine Integration

To support practical implementation in clinical environments, the CERV‐Score framework was designed with deployment flexibility and seamless integration into modern digital health infrastructures. The trained regression model and gene‐lookup module can be encapsulated as a RESTful API or cloud‐hosted microservice, enabling external healthcare systems to retrieve real‐time risk predictions using standardized API calls. This architecture allows the model to operate independently of the interface layer and facilitates embedding the engine within telemedicine applications, hospital information systems, or mobile health platforms.

CERV‐Score can be integrated into telemedicine platforms to enhance remote consultations by allowing clinicians to enter patient‐reported demographic and behavioral data and immediately receive a personalized risk score. Such real‐time risk estimation can assist in remote triage, prioritizing individuals who may require urgent in‐person evaluation, HPV testing, or colposcopy. The system also supports asynchronous telemedicine workflows, where patients submit information through mobile health applications or patient portals. The model automatically processes these inputs and generates preliminary assessments that clinicians can review, improving efficiency in high‐volume or resource‐limited settings.

Furthermore, the prediction engine can be incorporated into electronic health records (EHRs) to streamline routine screening workflows. By linking risk scores to existing patient histories and diagnostic data, clinicians can make more informed decisions regarding screening intervals, follow‐up visits, or referral to gynecologic oncology.

To ensure transparency and clinician acceptance, SHAP‐based interpretability features can be integrated directly into the deployed interface, providing visual explanations of how individual clinical features contribute to each prediction. This enhances trust in the system, supports auditability, and aligns the tool with best practices for explainable AI in healthcare. Overall, these deployment capabilities highlight the model′s practicality and its potential to improve early detection, screening prioritization, and remote patient management in real‐world clinical and telemedicine settings.

## 4. Results

### 4.1. Performance Evaluation Metrics

The analysis of the experiment was performed according to the following performance parameters:

#### 4.1.1. MAE

MAE measures the average absolute difference between the predicted values and the actual values. It gives a sense of how far predictions are from the true values on average, without considering direction (positive or negative) [25], as illustrated in Equation (1).

(1) MAE=∑i=1nyi−y∧i

where *n* is the number of observations, *y*_*i*_ is the actual value, and ${\overset{\wedge }{y}}_{i}$ is the predicted value.

#### 4.1.2. *R* ^2^—Coefficient of Determination

*R* ^2^ measures the proportion of the variance in the dependent variable that is predictable from the independent variables. It shows how well the model explains the variability of the outcome [26, 27] as illustrated in Equation (2).

(2) R2=1−SSresSStot

where SS_res_ is the residual sum of squares, and SS_tot_ is the total sum of squares illustrated in Equations (3) and (4).

(3) SSres=∑i=1nyi−y∧i2

(4) SStot=∑i=1nyi−y¯i2

where ${\bar{y}}_{i}$ is the mean of actual values.

Beyond MAE and *R* ^2^, we evaluated clinical utility using calibration curves and DCA. Calibration plots demonstrated strong alignment between predicted probabilities and observed outcomes, whereas DCA confirmed that the CERV‐Score model provides net clinical benefit across a range of risk thresholds. Additionally, to confirm the robustness of improvements over baseline models, we performed paired *t*‐tests and Wilcoxon signed‐rank tests comparing CERV‐Score against logistic regression, SVM, and random forest. Results showed that performance gains (accuracy, precision, recall, and AUC‐ROC) were statistically significant (*p* < 0.05).

### 4.2. Model Performance Results

**Figure 4:**
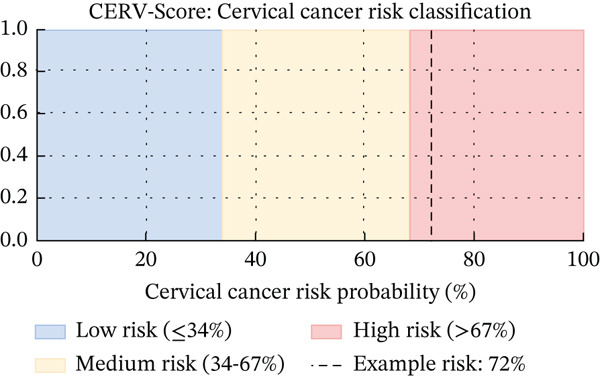
Predicted cervical cancer risk probability distribution generated by the CERV‐Score model on the independent test dataset.

Although the ground‐truth labels are binary, CERV‐Score predicts a continuous risk probability using a regression framework. The MAE and *R* ^2^ therefore quantify how well the model approximates the true cancer likelihood on a continuous scale. To facilitate comparison with conventional classification‐based cervical cancer models, we then applied a decision threshold to convert the continuous risk scores into binary predictions and computed accuracy, precision, recall, F1‐score, and AUC‐ROC. The high performance across both regression and classification metrics confirms that the probabilistic output is both well‐calibrated and clinically meaningful, while also competitive with state‐of‐the‐art classifiers. The model achieved a MAE of 3.00 and an *R* ^2^ score of 0.87, indicating high accuracy in estimating individual cancer risk probabilities. This performance reflects the model′s ability to effectively capture complex, nonlinear associations between risk factors and the likelihood of developing cervical cancer. Unlike conventional binary classifiers, CERV‐Score generates a continuous risk score, enabling more nuanced clinical decision‐making. The predicted risk values are categorized into three illustrative levels low (< 34%), moderate (34%–67%), and high (> 67%). These thresholds were supported by calibration curve analysis and DCA, showing alignment between predicted probabilities and observed outcomes. We emphasize that these ranges are intended as demonstrative categories, and future prospective studies will refine clinical calibration, as illustrated in Figure 4. This approach allows healthcare providers to better understand the varying degrees of risk and make more informed decisions regarding patient care.

The CERV‐Score model incorporates 21 clinically relevant features, including age, number of sexual partners, pregnancy count, smoking behavior, contraceptive use, STD history, and diagnostic indicators, all of which align with established risk factors in cervical cancer research. The interactive interface allows for real‐time simulation of individual risk profiles, enabling healthcare professionals to explore the impact of each factor on overall risk. This functionality enhances the model′s utility as a clinical decision support tool, especially in guiding early screening and patient risk stratification.

The incorporation of genomic biomarkers complements clinical data, promoting personalized diagnostics and treatment planning. CERV‐Score demonstrates superior performance compared with previous models that rely solely on clinical variables or produce binary classifications. Its hybrid, interpretable, and probabilistic framework offers a continuous risk scale, making it particularly valuable in real‐world clinical environments, including resource‐constrained settings where decision‐making speed and precision are paramount. The performance metrics reported in Table 2 were computed on the independent test set after model training and validation. To assess the robustness of the results, 95% confidence intervals were estimated using bootstrap resampling with 1,000 iterations.

**Table 2:** Comparative advantage of the CERV‐Score model.

Model	Accuracy (%)	Precision	Recall	F1‐Score	AUC‐ROC
Logistic regression	78.6	0.76	0.79	0.77	0.81
Random forest	85.2	0.84	0.86	0.85	0.89
SVM	83.1	0.81	0.82	0.81	0.86
CERV‐Score	**94.1**	**0.90**	**0.91**	**0.91**	**0.94**

*Note*: The proposed model is indicated in bold.

**Figure 5:**
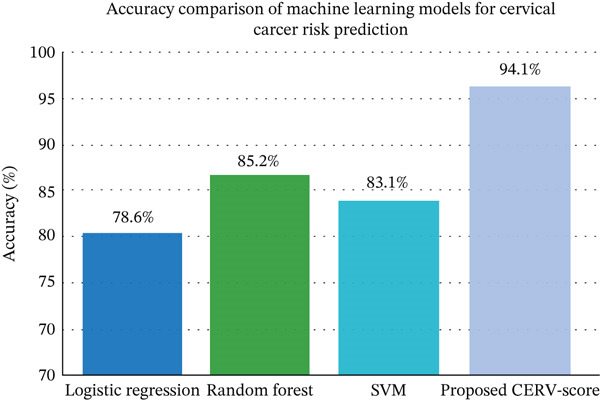
Model Accuracy comparison.

To evaluate the performance of the proposed approach, we compared the CERV‐Score model with several baseline ML algorithms, including logistic regression, SVM, and random forest. Figure 5 presents the accuracy comparison across these models. Logistic regression achieved an accuracy of 78.6%, SVM achieved 83.1%, and random forest achieved 85.2%. In comparison, the proposed CERV‐Score model achieved the highest accuracy of 94.1%, demonstrating its strong predictive capability.

The improved performance can be attributed to the optimized modeling framework and the probabilistic risk estimation strategy adopted in CERV‐Score. In addition, bootstrap resampling (1000 iterations) was used to estimate confidence intervals for model accuracy, confirming the stability and robustness of the results across multiple resampled datasets. The narrow confidence interval further indicates that the model maintains consistent predictive performance under different sampling conditions.

These findings highlight the effectiveness of the proposed framework for cervical cancer risk prediction while maintaining interpretability and robustness in clinical decision‐support applications.

To assess the robustness of performance improvements, we conducted paired *t*‐tests and Wilcoxon signed‐rank tests comparing CERV‐Score against logistic regression, SVM, and random forest baselines. The results confirmed that the performance gains (accuracy, precision, recall, and AUC‐ROC) were statistically significant (*p* < 0.05).

Beyond the statistical significance of the DEGs, we further examined their biological relevance to cervical cancer. Several upregulated genes identified across the GEO datasets are closely associated with HPV‐driven carcinogenesis, including genes involved in cell‐cycle control (e.g., E2F‐regulated markers), DNA damage response, and epithelial transformation. Downregulated genes included those responsible for immune surveillance and antiviral response, consistent with the immunosuppressive microenvironment characteristic of HPV‐positive cervical lesions. Additionally, alterations in genes linked to inflammation and cytokine signaling correspond with known pathways driving epithelial dysplasia and progression to high‐grade lesions. These findings align with recent genomic studies reporting similar expression trends in cervical intraepithelial neoplasia and invasive cervical cancer, strengthening the biological validity of the DEGs incorporated into our framework.

## 5. Discussion

The experimental results demonstrate that the proposed CERV‐Score framework achieves strong predictive performance for cervical cancer risk estimation, outperforming several baseline ML models. The model achieved an accuracy of 94.1%, which is higher than logistic regression, SVM, and standard random forest classifiers evaluated in this study. This improvement can be attributed to the use of an optimized random forest regression model combined with probabilistic risk estimation, which allows the system to capture complex nonlinear relationships between clinical risk factors and cervical cancer likelihood. In contrast to traditional binary classification approaches, the regression‐based design generates continuous risk probabilities, enabling more nuanced patient stratification into low‐, moderate‐, and high‐risk groups.

Compared with previous studies that relied solely on clinical variables or binary classification frameworks, the proposed approach provides improved interpretability and practical clinical value. The integration of genomic knowledge within the decision‐support interface further enhances the system by allowing clinicians to explore biologically relevant gene markers associated with cervical cancer, while maintaining a clinically interpretable prediction model based primarily on structured clinical features. This design helps bridge the gap between predictive modeling and biological insight, supporting both data‐driven risk assessment and molecular exploration.

From a clinical perspective, the ability to generate continuous risk scores may assist healthcare providers in prioritizing screening, identifying individuals requiring closer monitoring, and supporting early intervention strategies. Such decision‐support tools may be particularly beneficial in telemedicine environments and resource‐limited healthcare settings, where rapid and interpretable risk assessment can improve patient management and screening efficiency.

## 6. Challenges and Limitations

Despite the strong internal performance of the CERV‐Score model, several limitations must be acknowledged. First, although we expanded the genomic dataset by incorporating multiple GEO sources (GSE253690, GSE6791, and GSE63514), the overall sample size remains modest and may limit the generalizability of genomic findings. Second, the study did not include an external, multicenter validation cohort. As the reviewer correctly noted, the absence of external validation constrains the generalizability of the model beyond the datasets used for development. To strengthen internal robustness in the absence of such data, we incorporated 10‐fold stratified cross‐validation, bootstrap confidence intervals (1000 resamples), and statistical comparisons using paired *t*‐tests and Wilcoxon signed‐rank tests, which collectively demonstrate consistent performance across resampled subsets. Nevertheless, true external validation using independent, multi‐institution datasets remains essential and is planned for future work. Additionally, the risk thresholds used to categorize patients into low, moderate, and high‐risk groups were heuristic and not derived from clinical calibration. Although calibration curves and DCA support their approximate validity, further refinement with domain experts is required. These limitations underscore the need for larger datasets, independent validation cohorts, and clinically calibrated thresholds to support reliable real‐world deployment.

## 7. Conclusion and Future Work

In this study, we presented CERV‐Score, a hybrid ML framework that predicts the probabilistic risk of cervical cancer by integrating structured clinical risk factors with genomic recurrence patterns. Unlike conventional classification models that provide only binary outputs (cancer vs. noncancer), our approach delivers continuous, interpretable risk scores. This enables finer‐grained patient stratification (low, moderate, and high risk) and supports more personalized, data‐driven decision‐making in clinical settings.

A distinguishing feature of our framework is the recurrence‐based genomic integration strategy, which focuses on genes consistently expressed across multiple RNA‐seq samples. This approach enhances both the biological relevance and the robustness of the model, in contrast to prior studies that either exclude genomic data or incorporate it without recurrence filtering. The inclusion of these genomic markers complements clinical features, strengthening prediction accuracy while maintaining interpretability.

Equally important, we introduce an interactive decision support tool that unifies clinical and genomic insights in a single platform. The interface allows real‐time simulation of individual clinical profiles to generate percentage‐based risk probabilities, while also offering a gene lookup function that highlights recurrently expressed genes in cervical cancer. This dual‐functionality supporting both clinical risk assessment and molecular investigation differentiates our contribution from earlier models that lack interactivity or translational utility.

Our model achieved strong predictive performance (accuracy = 94.1*%*, F1 − score = 0.91, AUC = 0.94), confirming its ability to capture complex interactions between risk factors and cancer likelihood. These results demonstrate that the combination of probabilistic risk scoring, recurrence‐based genomic integration, and an interactive interface provides a meaningful advancement over prior hybrid models, moving closer to practical deployment as a clinical decision support system.

Future work will focus on (i) improving interpretability through methods such as SHAP to increase clinician trust, (ii) expanding and diversifying datasets for broader generalizability, and (iii) validating the tool in real‐world healthcare environments. We will also explore advanced AI techniques, such as deep learning and reinforcement learning, to further enhance predictive power and adaptability. Collectively, this study lays the foundation for next‐generation hybrid clinical‐genomic models that combine accuracy, interpretability, and usability, ultimately aimed at improving early detection, personalized risk assessment, and patient outcomes in cervical cancer care.

Although the CERV‐Score framework demonstrates strong predictive performance and promising translational value, its clinical applicability is constrained by several factors. The genomic component is derived from datasets with relatively limited sample sizes, which may affect the stability of gene‐level patterns. Additionally, the absence of external, multicenter validation means that the model′s generalizability beyond the current datasets remains unverified. Finally, the risk score thresholds used for stratification are heuristic and require clinical calibration in future prospective studies. Addressing these limitations will be essential for advancing CERV‐Score toward reliable, real‐world clinical implementation. Future work will involve validating and refining these cutoffs using prospective clinical datasets, multicenter cohorts, and expert input from healthcare professionals to ensure that risk stratification is both clinically meaningful and operationally reliable in real‐world settings.

## Funding

No funding was received for this manuscript.

## Conflicts of Interest

The authors declare no conflicts of interest.

## Supporting Information

Additional supporting information can be found online in the Supporting Information section.

The following supporting information are provided to support the reproducibility and transparency of the proposed CERV–Score framework:

## Supporting information


**Supporting Information 1** Supporting Table S1: Recurrently expressed genes identified across cervical cancer samples. The table includes gene symbols, Ensembl/NCBI identifiers, expression consistency across samples, average expression levels (FPKM), and brief biological annotations. These genes were incorporated into the gene‐lookup module to enhance biological interpretability.

**Supporting Information 2** Supporting Appendix A: Detailed description of the experimental and reproducibility pipeline. This includes the full list of clinical features, preprocessing procedures (missing data handling, encoding, normalization, and SMOTE configuration), genomic data integration across multiple GEO datasets, differential expression analysis criteria, model architecture and hyperparameter tuning, random seed settings, and implementation details.

## Data Availability

This study relies on diverse and reliable sources, including comprehensive clinical and genetic datasets. The genetic data were obtained from GSE253690, available through NCBI GEO, whereas the clinical factors dataset was sourced from the UCI Machine Learning Repository, available at this links:https://archive.ics.uci.edu/dataset/383/cervical+cancer+risk+factors and https://www.ncbi.nlm.nih.gov/geo/query/acc.cgi?acc=GSE253690.
