# Supplementary material for: CERV‐Score: A Hybrid Machine Learning Framework for Cervical Cancer Risk Prediction Using Integrated Clinical and Genomic Data
Source: Int J Telemed Appl. 2026 May 6;2026:9913421. doi: 10.1155/ijta/9913421 (PMC13145354; doi:10.1155/ijta/9913421)
Supplement: Supplementary file 1 — Supporting Information 1 Supporting Table S1: Recurrently expressed genes identified across cervical cancer samples. The table includes gene symbols, Ensembl/NCBI identifiers, expression consistency across samples, average expression levels (FPKM), and brief biological annotations. These genes were incorporated into the gene‐lookup module to enhance biological interpretability. [file IJTA-2026-9913421-s001.docx]

**Supplementary Table S1. Recurrently Expressed Genes in Cervical Cancer Samples (GSE253690)**

The table below lists the subset of genes identified as *recurrently expressed* (present in ≥2 out of 3 RNA-seq cervical cancer samples). These genes were incorporated into the CERV-Score framework to strengthen biological relevance and allow genomic validation within the interactive interface.

| **Gene Symbol** | **Ensembl/NCBI ID** | **Expression Consistency (No. of Samples Expressed In)** | **Average FPKM** | **Biological Notes** |
| --- | --- | --- | --- | --- |
| TP53 | ENSG00000141510 | 3/3 | 45.2 | Tumor suppressor; frequently mutated in cervical cancer |
| CDKN2A | ENSG00000147889 | 2/3 | 32.1 | Cell cycle regulator; HPV-related oncogenesis |
| MCM2 | ENSG00000170421 | 2/3 | 27.4 | DNA replication factor; marker of proliferation |
| … | … | … | … | … |
