# Supplementary material for: CERV‐Score: A Hybrid Machine Learning Framework for Cervical Cancer Risk Prediction Using Integrated Clinical and Genomic Data
Source: Int J Telemed Appl. 2026 May 6;2026:9913421. doi: 10.1155/ijta/9913421 (PMC13145354; doi:10.1155/ijta/9913421)
Supplement: Supplementary file 2 — Supporting Information 2 Supporting Appendix A: Detailed description of the experimental and reproducibility pipeline. This includes the full list of clinical features, preprocessing procedures (missing data handling, encoding, normalization, and SMOTE configuration), genomic data integration across multiple GEO datasets, differential expression analysis criteria, model architecture and hyperparameter tuning, random seed settings, and implementation details. [file IJTA-2026-9913421-s002.docx]

**Supplementary Appendix A. Reproducibility Details**

**In the final version of this study, the genomic component was constructed using multiple GEO datasets, not only GSE253690. Specifically, we integrated gene expression profiles from GSE253690, GSE6791, and GSE63514 to expand the genomic sample size, increase biological robustness, and reduce dataset bias. Differentially expressed genes (DEGs) were identified using DESeq2 and limma across these datasets, applying stringent statistical thresholds (adjusted p-value < 0.05 and |log2FC| > 1). This multi-dataset integration replaces earlier iterations of the work that relied solely on GSE253690 with three cancer samples and ensures consistency across the entire manuscript and supplementary materials.**

**A.1 Clinical Feature Set**

- Full list of 21 clinical variables (age, sexual partners, pregnancies, smoking, contraceptive use, STD history, etc.).

**A.2 Data Preprocessing Pipeline**

- Handling of missing values (“?” → NaN, imputation/removal rules).
- Encoding of categorical/binary features.
- Standardization of numerical features (StandardScaler).
- Class imbalance treatment using SMOTE (settings and ratios).

**A.3 Genomic Data Integration**

- RNA-seq normalization method: FPKM.
- Recurrence filter: ≥2 out of 3 cancer samples expressed.
- Incorporation into model and lookup tool.

**A.4 Model Configuration**

- Algorithm: Random Forest Regressor.
- Hyperparameters: number of trees, depth, minimum samples split, maximum features, etc.
- GridSearchCV ranges and final values.
- Train/test split ratio (80/20) and cross-validation details.

**A.5 Random Seeds**

- Seed values for data splitting, SMOTE oversampling, and model initialization to ensure exact reproducibility.

**A.6 Code Availability**

- Preprocessing scripts, training code, and interactive tool implementation will be provided in a **public GitHub repository** upon acceptance.
